# Nutrition and Dementia: Evidence for Preventive Approaches?

**DOI:** 10.3390/nu8030144

**Published:** 2016-03-04

**Authors:** Marco Canevelli, Flaminia Lucchini, Federica Quarata, Giuseppe Bruno, Matteo Cesari

**Affiliations:** 1 Department of Neurology and Psychiatry, “Sapienza” University, Rome 00185, Italy; flaminialucchini@hotmail.it (F.L.); federica.quarata@libero.it (F.Q.); giuseppe.bruno@uniroma1.it (G.B.); 2 Gérontopôle, Centre Hospitalier Universitaire de Toulouse, Toulouse 31062, France; macesari@gmail.com; 3 Université de Toulouse III Paul Sabatier, Toulouse 31062, France

**Keywords:** dementia, cognitive disorders, preventive strategies, randomized controlled trials, nutrition, diet

## Abstract

In recent years, the possibility of favorably influencing the cognitive trajectory through promotion of lifestyle modifications has been increasingly investigated. In particular, the relationship between nutritional habits and cognitive health has attracted special attention. The present review is designed to retrieve and discuss recent evidence (published over the last 3 years) coming from randomized controlled trials (RCTs) investigating the efficacy of nutritional interventions aimed at improving cognitive functioning and/or preventing cognitive decline in non-demented older individuals. A systematic review of literature was conducted, leading to the identification of 11 studies of interest. Overall, most of the nutritional interventions tested by the selected RCTs were found to produce statistically significant cognitive benefits (defined as improved neuropsychological test scores). Nevertheless, the clinical meaningfulness of such findings was not adequately discussed and appears controversial. In parallel, only 2 studies investigated between-group differences concerning incident dementia and mild cognitive impairment cases, reporting conflicting results. Results of the present review suggest that several dietary patterns and nutritional components may constitute promising strategies in postponing, slowing, and preventing cognitive decline. However, supporting evidence is overall weak and further studies are needed.

## 1. Introduction

The aging of our societies is leading to a dramatic increase in the prevalence of chronic conditions, threatening the sustainability of our healthcare systems. In particular, dementia is being increasingly recognized as a public health priority, given its enormous socioeconomic burdens in the absence of effective treatments [1]. In this context, the adoption of preventive strategies against dementia has repeatedly been solicited [2,3].

In the last decades, observational studies have indicated a wide range of potentially modifiable risk factors for dementia that could constitute targets for preventive strategies [4]. This has promoted a gradual shift of the scientific understanding of dementia from that of an unpreventable late-life condition to that of a lifelong disease process resulting from the competition of multiple risk and protective factors [5]. The most consistent evidence concerns vascular risk factors (e.g., hypertension, diabetes, obesity), psychosocial factors (e.g., depression), lifestyle behaviors (e.g., low mental and physical activity, smoking) [4,6]. High educational attainment and work complexity, social networking, engagement in mentally stimulating activities, and regular physical exercise have instead shown protective properties against dementia [4,6,7]. The promising epidemiological evidence suggesting a reduction of incident dementia [8] has been (at least partly) attributed to actions targeting cardiovascular risk factors (also through a more effective and wider use of specific treatments such as aspirin and lipid-lowering therapy) and increasing awareness. Based on these evidences, studies testing the efficacy of multidimensional interventions against dementia, mostly based on lifestyle modifications, have being increasingly designed and conducted [3].

In this scenario, a growing body of evidence has been focused on the association between dietary habits and cognitive performance/dementia. A recent meta-analysis of available cohort studies indicated that several dietary patterns and nutritional components (*i.e.*, Mediterranean diet, unsaturated fatty acids, antioxidants (such as vitamin E, vitamin C, and flavonoids, vitamin B)) are associated with a significantly reduced risk of dementia [9]. Furthermore, low concentrations of vitamin D have been related with an increased risk of cognitive decline [9]. Such nutritional components may exert their protective function in multiple and convergent ways. They have been shown to down-regulate the main pathophysiological pathways and processes linked to development of dementia (in particular, Alzheimer’s disease (AD)), including amyloid deposition, neurofibrillary degeneration, synapse loss, inflammation, increased oxidative stress, defects in mitochondrial function and cellular energy production, loss of vascular integrity, and neuronal injury [10]. Based on these considerations, the possibility of favorably influencing the cognitive trajectory by promoting the adoption of specific nutritional habits has been increasingly investigated.

Despite providing crucial information concerning risk profiles, observational studies reporting associations between nutritional patterns and dementia present many limitations: the dietary assessment usually completed only once before the outcome ascertainment; the heterogeneity of scores used to define dietary conformity as well as the cognitive evaluation; and the relevant residual confounding due to the major influence exerted by socioeconomic factors on nutritional habits [11,12]. To address these issues and advance in the field, multiple randomized controlled trials (RCT) testing nutritional interventions aimed at enhancing cognitive functioning in older persons have started to appear in the literature. Various nutritional compounds and dietary modifications (e.g., vitamins, omega-3 fatty acids, ketogenic diet, antioxidants) have been found to improve cognitive performance in patients diagnosed with dementia and AD [10].

In view of the growing interest on this topic, the aim of the present study is to provide a comprehensive, updated review of the recent literature (published over the past 3 years) exploring the relationship between nutrition and cognitive performance. In particular, we focused on RCTs exploring the efficacy of nutritional interventions at improving cognitive functioning and/or preventing dementia among non-demented (*i.e.*, cognitively healthy or presenting mild cognitive disturbances) older individuals.

## 2. Methods

### 2.1. Identification and Selection of Studies

**Figure 1:**
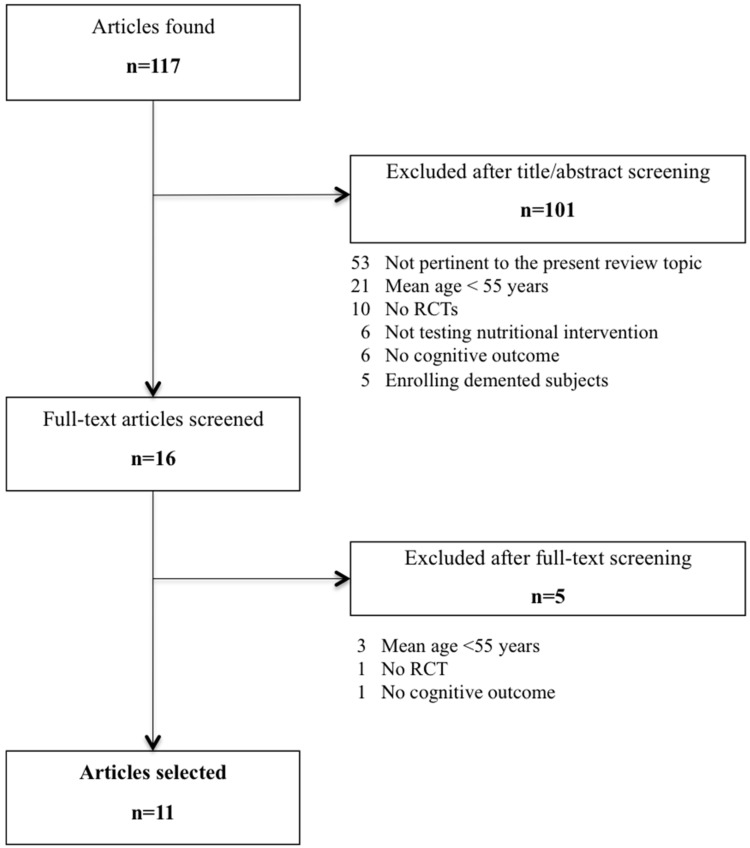
Flow-chart of articles selection.

The flowchart depicted in Figure 1 shows the process leading to the selection of the articles of interest for the present review. We performed a Medline literature search of studies published over the past 3 years (from 1 November 2012 to 31 December 2015) using the Medical Subject Heading (MeSH) terms “Randomized Controlled Trial”, “Human” and “English” combined with the following terms: (“diet*” OR “nutr*” OR “food” OR “aliment*”) AND (“cognit*” OR “dement*” OR “Alzheimer” OR “memory”). References of considered studies were also reviewed to identify further relevant publications. The identified articles were singularly evaluated according to the following inclusion criteria:

(1) reporting results from RCTs;
(2) testing nutritional interventions;
(3) adopting at least one outcome measure assessing cognitive performance and/or dementia (or mild cognitive impairment, MCI) incidence;
(4) enrolling non-demented (*i.e.*, cognitively healthy or presenting mild cognitive disturbances) older persons; and
(5) recruiting individuals with a mean age ≥55 years.

### 2.2. Data Extraction

For each study retained for the present review, Authors abstracted the following data: number of participants, demographic characteristics of the samples, characteristics of adopted interventions (*i.e.*, doses, duration), outcome measures (in particular, cognitive function modifications and/or incidence of dementia or MCI), main results and conclusions. Three Authors (Marco Canevelli, Flaminia Lucchini and Federica Quarata) discussed the collected data and reached a consensus to resolve the existing discrepancies.

## 3. Results

A total of 117 articles were retrieved from the literature to identify studies of potential interest for the present review (Figure 1). After a prescreening based on titles and abstracts, 16 articles were fully evaluated. Thus, 11 studies were finally selected [11,13,14,15,16,17,18,19,20,21,22]. The main reasons for articles’ exclusion were the non-RCT design, and the age of the sampled populations (<55 years). It is noteworthy that two of the included studies referred to the same study population (*i.e.*, the PREDIMED-NAVARRA study) [17,18]. We decided to retain both the studies as they described different analyses, performed in different subgroups of participants. Table 1 provides an overview of the included studies for what concerns the characteristics of participants, nutritional interventions, adopted cognitive outcomes, and main findings.

### 3.1. Study Samples

Overall, 2631 subjects were recruited and randomized in the retained studies, with sample sizes widely ranging between 37 and 1260 participants. The weighted mean age of the enrolled individuals was 68.7 (standard deviation, SD 7.6) years, varying between 55.8 and 79.0 years across studies. Two RCTs were gender-restricted, recruiting only healthy older women [13] and healthy elderly male volunteers [14], respectively. Five studies specifically targeted subjects at risk for diverse adverse outcomes. In particular, two studies [21,22] enrolled pre-frail and frail participants according to the Fried and colleagues’ operationalization of frailty [23]. Two studies focused on older individuals at high vascular risk (*i.e.*, presenting diabetes or ≥3 vascular risk factors) [17,18]. Finally, one study [19] recruited subjects at risk for cognitive decline, as indicated by high scores at a dementia risk index [24]. Noticeably, in two studies, the exclusion of participants already exhibiting an overt dementia was not explicitly described [21,22]. Nevertheless, the high mean MMSE scores of the recruited samples indicated a normal or just mildly impaired global cognitive performance.

### 3.2. Nutritional Interventions

In nearly one third of the retained RCTs, the nutritional intervention was delivered in the context of a multidomain strategy including also strength training [13], resistance-type exercise [21,22], or, more broadly, a combination of physical activity, cognitive training, and vascular risk monitoring [19]. Overall, eight different interventions were tested. Seven studies explored the efficacy of single, specific dietary components such as creatine [13], flavanols [15,16], milk protein concentrates [21,22], polyunsaturated fatty acids [20], and a herbal food substance (*i.e.*, *Nigella sativa* Linn. or black cumin) [14]. On the other hand, the remaining four RCTs assessed the cognitive benefits produced by more complex dietary regimens, such as the Mediterranean diet (supplemented with extra virgin olive oil or mixed nuts) [11,17,18] and a similar pattern developed in accordance to the Finnish Nutritional recommendations [19]. The adherence to both of these diets was promoted through individual and group counseling sessions (mostly leaded by nutritionists), providing practical information and support for facilitating lifestyle and dietary changes. In the PREDIMED studies [11,17,18], written material including descriptions of seasonal foods, shopping lists, weekly meal plans, and cooking recipes was also provided. Similar recommendations were given to the participants of these three studies: high consumption of vegetables, fruits, legumes and cereals, olive oil as the principal source of monounsaturated fat, low intake of saturated fat, moderate intake of fish, low-to-moderate intake of dairy products, low consumption of meat and poultry, and wine consumed in low-to-moderate amounts. These three RCTs tested the cognitive efficacy of such nutritional strategies over the long term, with the duration of the adopted interventions ranging between 2 and 6.5 years. The other studies (testing single components) had instead a shorter duration (ranging between 5 and 24 weeks).

### 3.3. Cognitive Outcomes and Main Findings

Only three of the retained studies adopted the cognitive status of participants as outcome variable, investigating the incidence of MCI and dementia after 6.5 [17,18] and 4.1 [11] years of intervention (consisting in Mediterranean diet for both of the studies), respectively. Nevertheless, only in two of them between-groups differences in the observed MCI/dementia incidence were specifically investigated. In the first study [17], a lower incidence of MCI was observed among subjects randomized to Mediterranean diet plus extra virgin olive oil compared to the control group (odds ratio, OR: 0.34; 95% CI: 0.12–0.97). In the other study [11], the occurrence of MCI was found to be similar in the two randomization groups. No significant between-group difference concerning incident dementia cases was noticed in both studies.

The other RCTs adopted diverse neuropsychological measures and tools as primary outcomes, thus assessing pre-post intervention modifications of cognitive scores. Most of these studies used comprehensive, standardized batteries, consisting of various tests specifically targeting memory (both verbal and visual), attention and executive functions, visuo-spatial abilities, processing speed, and language skills. Only one study exclusively focused on a single cognitive aspect, that is object recognition [15]. Overall, nearly all the tested interventions were found to be effective at improving the targeted neuropsychological functions, resulting in a significant improvement of global cognitive functioning, memory tasks, language skills, executive functioning, and processing speed. Consistently, post-hoc analyses of the FINGER study showed that the risk of overall and domain-specific (*i.e.*, executive functions and processing speed) cognitive decline was higher in the control group than in the intervention group [19]. One study reported negative findings from creatine supplementation [13].

## 4. Discussion

The present review was aimed at retrieving and discussing recent evidence from RCTs investigating the efficacy of nutritional interventions in improving cognitive functioning and/or preventing cognitive decline among non-demented older individuals.

A growing interest has been recently attracted by the possible relationship existing between nutrition and cognitive health, leading to the design and conduction of several interventional studies. Nevertheless, the evidence suggesting a potential preventive effectiveness of nutritional strategies towards cognitive disorders is still scarce. Only two out of the eleven retained studies [11,17] provided data concerning the possibility of actually preventing/delaying cognitive disorders among older individuals by implementing a nutritional intervention (*i.e.*, Mediterranean diet). These RCTs also produced somehow conflicting results, being the MCI incidence found to be lower in the treatment group in one study [17], while similar in the treatment and control groups in the other one [11]. Moreover, no significant difference concerning incident dementia cases was observed in the two studies. The paucity of evidence on this topic, as well as the disproportion between observational and experimental data in the field, is likely to be referred to the difficulty in performing preventive RCTs against cognitive disorders (and more generally, against age-related pathological conditions [25]). Several methodological issues (e.g., difficulty at selecting the target population, the precarious balance between optimal timing of the intervention and incidence of the primary outcome, and the need of long follow-up and large sample sizes [26]) have been frequently encountered in the conduction of such researches resulting, to date, in an almost complete lack of substantial evidence about dementia prevention. It is noteworthy that additional useful information might be shortly provided by ongoing international RCTs testing the cognitive efficacy of multidomain, long-lasting interventions (including also diet and nutritional advices) [19,27,28].

Most of the selected studies considered the changes in cognitive and neuropsychological performance as primary outcome. In most of cases, the tested interventions produced a statistically significant improvement of cognitive measures. These findings appear to be mostly confirmatory of available evidence coming from observational studies, indicating that the exposition to specific nutritional compounds (e.g., flavonoids, unsaturated fatty acids) or dietary patterns (e.g., Mediterranean diet) may result in cognitive benefits [9]. Nevertheless, the clinical meaningfulness of such results was not at all discussed. None of the retained articles explicitly questioned if the measured cognitive benefits (in terms of improved cognitive scores) could have been actually responsible for a real clinical benefit (in terms of personal wellbeing and functioning). Given the challenges in adopting “hard outcomes” (such as dementia incidence) in preventive trials, the use of composite scores including several validated cognitive tests has been recommended [29]. Nevertheless, the clinical significance of the observed cognitive changes still needs to be clarified [26]. This aspect appears to be particularly challenging when healthy or mildly impaired participants are recruited. Findings from selected RCTs, beside the ambiguous personal and clinical relevance, may be thus more appropriately interpreted in a public health context, in which small long-term effects on common disorders could have high relevance [26].

Interestingly, several studies included in the present review focused on individuals at risk of cognitive decline and dementia (being frail or at high vascular risk, or exhibiting high scores at dementia risk scores). Moreover, in nearly half of these studies, the tested nutritional interventions were part of broader strategies simultaneously targeting various domains of the older person (*i.e.*, physical and mental activity, vascular care). These two approaches (*i.e.*, the identification of at-risk populations and the realization of multidomain interventions) have been repeatedly advocated in the field of dementia prevention [26]. Restricting the focus on those individuals that are more likely to develop dementia could increase the statistical power (thus allowing smaller sample size) and avoid unnecessary interventions in subjects at low risk. In parallel, the adoption of multi-component interventions (despite rendering difficult to determine which of the targeted components is responsible for the observed benefit) could consent to more properly target the diverse factors contributing to overall dementia risk. 

Another aspect potentially limiting the implementation of dementia preventive strategies is represented by their feasibility in the “real world”. None of the selected studies provided detailed information concerning the transferability (e.g., in terms of costs, resources, potential socio-cultural barriers) of the experimental interventions. At the same time, it should be noticed that nutritional actions relying on healthy dietary patterns (such as the Mediterranean diet and similar regimens) have already been shown to be cost-effective and easy-to-implement [30]. These aspects, combined with the observed benefits produced by such nutritional strategies, are contributing to a gradual shift in nutritional epidemiology from a “single nutrient” approach to a “whole diet” paradigm, more broadly aimed at capturing the cumulative effects of the overall diet on the health status of the individual [12].

## 5. Conclusions

The possibility of postponing or preventing dementia through nutritional and dietary habits has been increasingly investigated. In line with available epidemiological evidence, several nutritional compounds and regimens have shown to produce significant cognitive benefits among older persons enrolled in placebo-controlled studies. Nevertheless, despite these encouraging findings, robust supportive evidence is still lacking. In particular, further RCTs with long follow-up are needed in order to determine whether specific nutritional components or patterns may reduce the occurrence of cognitive disorders and overt dementing illnesses. In parallel, the clinical meaningfulness of the observed cognitive benefits, as measured by neuropsychological score changes, should be properly addressed and discussed.

## Figures and Tables

**Table 1:** Randomized controlled trials exploring the cognitive effects of nutritional interventions among non-demented older individuals published over the last 3 years.

Reference	Study Sample	Intervention(s)	Duration	Cognitive Outcome(s)	Main Results
Alves *et al.* 2013 [13]	*n* = 56 healthy older women (mean age 66.8 years)	(1) Creatine (20 g/day for 5 days, then 5 g/day) (2) Creatine + strength training (3) Placebo (4) Placebo + strength training	24 weeks	MMSE; Stroop test; TMT; Digit Span; Delayed recall test	Creatine supplementation did not promote any significant cognitive benefit
Bin Sayeed *et al.* 2013 [14]	*n* = 40 healthy elderly males (mean age 55.8 years)	(1) *Nigella sativa* Linn. Seeds (1000 mg/day) (2) Placebo	9 weeks	WMS; Digit Span; ROCF; LCT; TMT; Stroop test; Logical memory test	Significant improvement of all the cognitive scores in the *Nigella sativa* group
Brickman *et al.* 2014 [15]	*n* = 37 healthy, sedentary older subjects (mean age 57.7 years)	(1) High flavanol intake (900 mg cocoa flavanols and 138 mg of (−)-epicatechin/day) + exercise (2) High flavanol intake (3) Low flavanol intake (10 mg cocoa flavanols and <2 mg (−)-epicatechin/day) + exercise (4) Low flavanol intake	12 weeks	ModBent task	A high-flavanol intervention had a significant effect on ModBent performance, independent of exercise
Kean *et al.* 2015 [16]	*n* = 37 healthy older subjects (mean age 66.7 years)	(1) High flavanone drink (305 mg/day) (2) Low flavanone drink (37 mg/day)	8 weeks	CERAD; SWM; DSST; LM; Go-NoGo; Letter Fluency; Serial sevens; WMS	Significant improvement of global cognitive function in the high flavanone group
Màrtinez-Lapiscina *et al.* 2013 [17] *	*n* = 268 older subjects at high vascular risk (mean age 74.1 years)	(1) MedDiet + EVOO (1 L/w) (2) MedDiet + mixed nuts (30 g/day) (3) Control diet (advice to reduce dietary fat)	6.5 years	MMSE; CDT; WMS; FAS; RAVLT; ROCF; BNT; CDR; TMT; WAIS; Digit span Cognitive status	Significant improvement of fluency and memory tasks in MedDiet + EVOO group. Reduced MCI incidence
Màrtinez-Lapiscina *et al.* 2013 [18]	*n* = 522 older subjects at high vascular risk (mean age 67.4 years)	(1) MedDiet + EVOO (1 L/week) (2) MedDiet + mixed nuts (30 g/day) (3) Control diet (advice to reduce dietary fat)	6.5 years	MMSE; CDT	Significant improvement of cognitive performance in the two MedDiet groups
Ngandu *et al.* 2015 [19]	*n* = 1260 older subjects at high risk of cognitive decline (mean age 69.3 years)	(1) Diet (Finnish Nutrition Recommendations) + exercise + cognitive training + vascular risk monitoring (2) General health advice	2 years	Comprehensive neuropsychological test battery (CERAD)	Significant improvement of global cognition, executive functioning and processing speed
Nilsson *et al.* 2012 [20]	*n* = 40 healthy older subjects (mean age 63.3 years)	(1) Fish oil n-3 PUFA (3 g/day) (2) Placebo	5 weeks	Working memory and selective attention tests	n-3 PUFA intervention significantly improved working memory
Valls-Pedret *et al.* 2015 [11]	*n* = 447 cognitively healthy older subjects (mean age 66.9 years)	(1) MedDiet + EVOO (1 L/week) (2) MedDiet + mixed nuts (30 g/day) (3) Control diet (advice to reduce dietary fat)	4.1 years (median)	MMSE; WMS; RAVLT; WAIS; CTT; FAS; Digit span Cognitive status	Significant improvement of all the cognitive functions in the 2 MedDiet groups. No difference in MCI incidence
van de Rest *et al.* 2014 [21]	*n* = 127 frail or pre-frail older subjects (mean age 79 years)	(1) Protein (30 g/day) (2) Protein + exercise (3) Placebo (4) Placebo + exercise	24 weeks	MMSE; TMT; Stroop test; WMS; WLT; VFT; Reaction time tasks; Digit span	Exercise training in combination with protein supplementation improved information processing speed
van der Zwaluw *et al.* 2014 [22]	*n* = 65 frail or pre-frail older subjects (mean age 79 years)	(1) Protein (30 g/day) (2) Placebo	24 weeks	MMSE; TMT; Stroop test; WMS; WLT; VFT; Reaction time tasks; Digit span	Improvement of reaction time in the protein supplementation group

* The study reports results observed in a subgroup of the population enrolled in the study [18]. EVOO: extra virgin olive oil; MedDiet: Mediterranean diet; PUFA: polyunsaturated fatty acids. Cognitive functions/domains assessed by the adopted cognitive tools and measures: Boston Naming Test (BNT): naming and animals fluency; Clinical Dementia Rating (CDR): dementia severity; Consortium to Establish a Diagnosis of Alzheimer’s Disease (CERAD): comprehensive neuropsychological test battery; Clock Drawing Test (CDT): global cognition; Color Trail Test (CTT): attention and visuomotor speed; Digit Span: working memory; Digit Symbol Substitution Test (DSST): working memory; FAS: semantic and phonemic fluency; Go-NoGo: inhibition and sustained attention; Letter Cancellation Test (LCT): visual attention; Letter Memory Task (LM): executive functions; Mini Mental State Examination (MMSE): global cognition; ModBent: object recognition; Rey Auditory Verbal Learning Test (RAVLT): immediate and delayed verbal memory; Rey-Osterrieth Complex Figure (ROCF): immediate and delayed visual memory; Spatial Working Memory (SWM): spatial working memory; Stroop test: selective attention; Trail Making Test (TMT):attention and executive functions; Verbal Fluency Test (VFT): semantic memory and language; Wechsler Adult Intelligence Scale (WAIS): executive functions; Wechsler Memory Scale (WMS): episodic memory; Word Learning Test (WLT): immediate and delayed verbal memory.
